# A realist evaluation of a novel cervical cancer prevention strategy in Iquitos, Peru

**DOI:** 10.1371/journal.pgph.0004517

**Published:** 2025-11-19

**Authors:** Valerie A. Paz-Soldan, Lauren Nussbaum, Joanna Brown, Graciela Meza-Sanchez, Sarah D. Gilman, J. Kathleen Tracy, Patti E. Gravitt

**Affiliations:** 1 Department of Tropical Medicine and Infectious Disease, Tulane University Celia Scott Weatherhead School of Public Health and Tropical Medicine, New Orleans, Louisiana, United States of America; 2 Asociación Benéfica Prisma, Lima, Peru; 3 Facultad de Medicina Humana, Universidad Nacional de la Amazonía Peruana, Iquitos, Peru; 4 School of Medicine and Health Sciences, George Washington University, Washington, District of Columbia, United States of America; 5 Larner College of Medicine, University of Vermont, Burlington, Vermont, United States of America; 6 Department of Epidemiology and Public Health, University of Maryland, Baltimore, Maryland, United States of America; PLOS: Public Library of Science, UNITED STATES OF AMERICA

## Abstract

Proyecto Precancer is an implementation science and systems-thinking project with the objective of facilitating the adoption of a new screen-and-treat intervention for cervical cancer prevention in Iquitos in the Peruvian Amazon basin. This intervention introduced human papillomavirus molecular testing and shifted treatment from the tertiary level to the primary level through visual assessment for treatment and thermal ablation for those eligible. To inform how we scale up this pilot project to new regions in Peru in collaboration with the Ministry of Health, we conducted this realist evaluation to learn what worked in our pilot intervention in Iquitos, in what circumstances, for whom, and why. We developed four initial program theories, tested them through interviews with 32 stakeholders, we refined the four program theories based on the interviews, and added a fifth theory. This evaluation revealed that continuous engagement with providers in a horizontal manner across the systems’ professional hierarchies (Program Theories 1, 4, and 5) and centering patients with a more convenient and accessible continuum of care can, ultimately, lead to improved screening and treatment rates and reduced patient loss to follow-up (Theories 2 and 3). Furthermore, we learned from our collaborators that embedded research within the public health system had high potential for sustainability due to local ownership. These insights will inform our work as this project assists the Ministry of Health in adapting and scaling up the intervention in other parts of Peru.

## Introduction

### Cervical cancer in Peru

Cervical cancer is the third most common cancer among women in Latin America and the Caribbean [1]. In the state of Loreto in the Peruvian Amazon, cervical cancer is the primary contributor to cancer-related deaths among women, and the mortality rate from cervical cancer in this state is the highest in Peru, at approximately 28.2 per 100,000 in 2022 [2].

The World Health Organization has called for the large-scale elimination of cervical cancer through human papillomavirus (HPV) vaccination, screening eligible women, and providing treatment for those who need it [3,4]. Cervical cancer early detection and treatment (EDT) programs play a crucial role in enabling health systems to reach the goal of cervical cancer elimination [5]. However, socioeconomic inequalities, limited education, lack of health insurance, and poor access to care, particularly follow-up care for abnormal results, all contribute to major barriers in some settings, leading to wide differences in screening coverage across countries [6,7]. As a result, EDT programs must consider and adapt to the contexts of each health system to reach screening and treatment targets.

### Study setting

Iquitos is the capital city of the Peruvian state of Loreto in the Northeast Amazon rainforest basin bordering Ecuador, Colombia, and Brazil. With a population of approximately 465,455 people [8], Iquitos is the largest city in the world that is accessible only by air or river [9]. Within Iquitos, transportation routes include networks of paved and unpaved roads that connect communities to each other, but throughout Loreto, there is only one main paved highway that extends approximately 100 km; most travel is by boat on the Amazon river and its tributaries.

The Peruvian health care system has three main sectors—the public Ministry of Health system; the semi-public, employer-based system; and the private system—each independently organized and administered, with its own health facilities and laboratories [7]. The Ministry of Health’s Seguro Integral de Salud (SIS) is the public healthcare insurance program that provides insurance to Peruvians living in poverty, who constitute 67% of the population in Loreto [10]. This intervention, described below, was piloted in the Micro Red Iquitos Sur (MRIS) health network, the largest SIS network in Iquitos [11]. The MRIS covers a population of 150,000 people, including a target population of 20,000 women 30–49 years of age eligible for HPV testing, covering terrain accessible by roads and boats, and is comprised of 19 health facilities (including two hospitals), categorized by the following tiers of care (Table 1):

**Table 1:** MRIS Health System Facilities and Representation in Study.

Level of Care	Description	Number of Facilities	Number of Facilities in Sample	Number of Participants in Sample Affiliated with Level of Care
**III**	Tertiary Level Hospital	1	1	6
**II**	Secondary Level Hospital	1	1	1
**I-4**	Health Center with Inpatient Facility	1	1	7
**I-3**	Health Center without Inpatient Facility	2	2	3
**I-2**	Health Post with a General Practitioner	8	5	9
**I-1**	Health Post without a General Practitioner	6	2	2
**TOTAL**		19	12	28

### Proyecto precancer

Proyecto Precancer (PPC) is an implementation science and systems-thinking project with the objective of facilitating adoption of a new screen-and-treat (SAT) intervention for cervical cancer EDT in the MRIS health network. PPC is a joint effort between researchers (from Peru and the United States) and over 90 health system stakeholders across various levels of the national, regional, and local Peruvian public health system. The research team placed participatory action research at the core of all activities, guiding everything from identifying system barriers to making decisions about strategies to undertake [11]. Our local stakeholders played a dual role in the implementation process in Iquitos. During Phase I, they participated as research subjects, offering essential insights into system processes, values, and perspectives. Their contributions were key to developing a shared, visual understanding of how the EDT system functioned in practice, not just in theory, within their specific context. As equal collaborators in phase II, stakeholders in the MRIS discussed evidence-based options and elected to adopt an HPV-based screen-and-treat (SAT) intervention, managing that implementation with the approval of the Regional Ministry of Health, with PPC acting as facilitator.

More specifically, the components of the intervention were:

1. Transitioning the cervical cancer screening approach in the MRIS from Pap smears and/or Visual Inspection with Acetic Acid (VIA) to HPV molecular testing, with the option of self-sampling (see [5,12] for descriptions of the prior Pap/VIA-based screening system);
2. Task-shifting treatment for women with positive HPV tests from the hospital level to the primary level, with a general practitioner performing visual assessment for treatment (VAT) for all women who tested positive for HPV, and providing thermal ablation (TA) treatment for those eligible (i.e., women with no acetowhite lesions or lesions covering less than 75% of transformation zone) during that same visit (see [13] for description of obstacles to treatment completion at the hospital level);
3. Referring those who were ineligible for TA for a colposcopy and/or clinical management with a gynecologist at one of the two hospitals in Iquitos; and
4. Developing a hybrid paper/electronic screening registry.

The implementation process for the intervention (described in greater detail in Gilman 2024 [14]) included the following actions taken by the research team:

1. Generating a detailed and visual EDT system map through stakeholder interviews and system audits to enable a shared stakeholder understanding of the current system;
2. Identifying and elevating local program champions who emerged as key stakeholders who were interested and engaged in all processes and critical to solving local challenges;
3. Finding leverage for change through group model building [15] workshops facilitated by PPC that employed deliberative dialogue [16] and scenario analysis of several evidence-based EDT approaches to test assumptions in real time, using a collaborative, exploration-focused approach that helped to bring about a shared vision for future implementation, which led local and regional health authorities and providers to choose the HPV-and VAT for TA-based SAT intervention described above;
4. Developing stakeholder-designed implementation plans, including adapting an internal hybrid data registry and monitoring and evaluation system to evaluate impact, working with authorities to add new services and codes to the public health billing system, adapting laboratory space for sample processing, helping the health system prepare for task-shifting, and training health care providers, all by bundling discrete implementation strategies to meet stakeholder needs across the implementation continuum (acceptability, adoption, implementation, scale-up, and maintenance); and
5. Establishing ongoing monitoring and evaluation tools using stakeholder-accepted outcomes, reviewing monitoring and evaluation results with stakeholders, and adapting the intervention where needed (e.g., when HPV screening increased significantly in urban health facilities but not in rural health facilities, stakeholders planned community campaigns in rural areas, where health providers approached women in their homes to offer them the opportunity to be screened for HPV via self-sampling).

The intervention resulted in HPV screening rates surpassing the WHO’s 70% monthly coverage targets within 6 months of implementation (70% target metric translated to 233 women/month using the Ministry of Health’s screening guideline of HPV testing every five years), compared to 31% who were screened regularly under the prior Pap smear- and VIA-based system (70% target metric translated to 390 women/month using the Ministry’s screening guideline of Pap smears or VIA every three years) [14]. In terms of treatment, 67.4% of HPV-positive women reached a completion of care endpoint within 6 months, compared with 30.2% of VIA-positive women at hospital facilities [14]. Quantitative findings related to intervention metrics are being analyzed and will be disseminated in a separate publication.

The pilot program in Iquitos ended in 2021, but the MRIS health network adopted and continues to implement the HPV- and TA-based screen-and-treat approach. Based partly on evidence generated through this pilot, the Peruvian Ministry of Health expanded implementation of the HPV-based screen-and-treat approach with thermal ablation nationwide. Specifically, the Ministry of Health invested in the Roche’s cobas® HPV molecular testing system as its primary cervical cancer screening technology; it has also updated the national cervical cancer screening and management guidelines to recommend HPV as the primary screening method and permit the TA-based SAT approach when other diagnostic and treatment tools are unavailable to mitigate loss to follow-up of HPV-positive patients. [17] PPC and the Ministry of Health signed a series of agreements in 2018 and 2022 which, among other things, memorialize PPC’s commitment to providing technical assistance as the Ministry of Health implements and scales up its HPV-based screen-and-treat (or screen-triage-treat) program, support improvements to Peru’s national data registry, and share the latest scientific evidence.

### Realist evaluation theory

Realist evaluation is a theory-based approach to program evaluation that goes beyond measuring a program’s outcomes (i.e., was the program successful), to understanding the underlying context and mechanisms that led specific outcomes to emerge (i.e., how and why and for whom was the program successful or not) [18,19]. The aim of practice-based evidence-generation is not to control for differences, but rather to *understand* them. Realist evaluation uses a phased implementation process: (1) formulation of initial program theories, (2) development of hypotheses summarizing what works, for whom, and why, (3) test of hypotheses with stakeholders, and (4) analysis and refinement of program theories [18].

Given the complexity of the fragmented and under-resourced Peruvian health system [20], particularly the interplay between the regional and national Ministry of Health in a semi-decentralized public health system [21], as well as the gap between how the system is supposed to work per Ministry of Health guidelines and how it actually works based on available resources [5,22], tracing the mechanisms behind PPC’s success would have been challenging using traditional evaluation methods.

To inform how to best provide technical assistance to regional cancer coordinators as they adopt, adapt, and scale up the Ministry of Health-driven HPV-based screen-and-treat approach to their regions in Peru, PPC conducted this realist evaluation to learn what worked in our cervical cancer EDT pilot in Iquitos, in what circumstances, for whom, and why. In other words, which elements of PPC’s collaboration with MRIS stakeholders most effectively supported their understanding of system needs and the implementation of the new approach? This realist evaluation will inform how PPC tailors its assistance in other regions in Peru and contribute to the emerging realist evaluation literature in resource-poor settings [23] to facilitate context-adaptation and adoption of similar HPV-based SAT strategies in other settings with similar systemic barriers.

## Methods

### Ethics statement

Ethics approval for this study was obtained from the institutional review boards at: Asociación Benéfica Prisma (CE0251.09), the Tulane University Biomedical IRB (16–891039IAA), Hospital Regional Loreto (ID-002-CIEI-2017), and Hospital Iquitos (065-ID-ETHICS COMMITTEE HICGG-2018). As approved by these IRB committees, all participants provided verbal and written informed consent to participate.

### Development of initial program theories

We (VPS, PG, KT, GM, JB, SG) developed four initial program theories based on three main processes: 1) weekly research team discussions over the course of seven months, 2) research team notes organized according to the Theoretical Domains Framework (TDF) [24] to structure the team’s reflections on the MRIS pilot implementation, which SG deductively coded using TDF; and 3) a focus group of 14 health care providers involved in the implementation of the SAT program throughout the health network to inform the development of the initial program theories. These 14 health care providers were purposively chosen to participate in the focus group because they were the key local collaborators in implementing the intervention; VPS and JB recruited these participants and conducted the focus group from September 15 through September 30, 2022.

**Fig 1:**
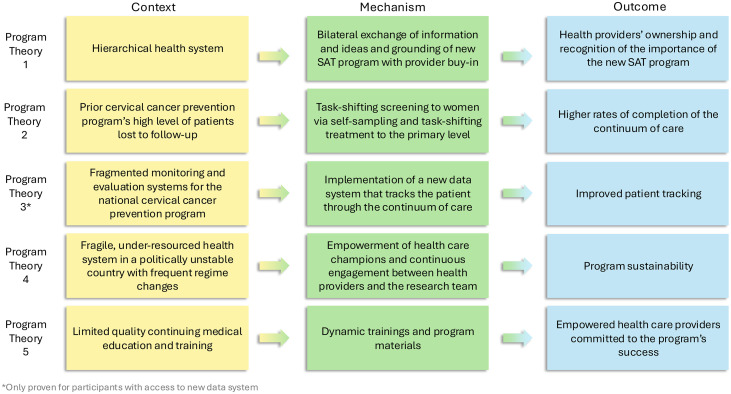
Program Theories.

The initial program theories were based on context-mechanism-outcome configurations that outlined patterns and causal chains pursuant to the realist evaluation framework (see Fig 1 below, in Results section).

### Participant selection and interview procedures

VPS, GM, JB, all Peruvian Spanish-speakers, then tested and iteratively refined the four initial program theories through 34 semi-structured, in-depth interviews in Spanish (S1 Text) to elicit the participants’ perspectives on the PPC pilot and to determine whether they agreed with the initial program theories [25]. We purposively sampled 34 participants who were health providers (28) and health authorities (5) from various levels of the health system, along with the dean of the national medical school in Loreto (1), all of whom were involved in the PPC pilot as local stakeholders (Table 2). These participants were recruited and interviewed in Iquitos from May 1 through June 27, 2023, and in Lima from August 1 through August 27, 2024. We began our interviews by asking open-ended questions to respondents about their own reflections regarding which elements of the intervention were most helpful and which could be improved so that respondents had the opportunity to provide their own input regarding the program theories without being driven by our initial theories (S1 Text, S2 Text).

**Table 2:** Study Participants.

Study Participants	Number (n = 34)
**Type of Health Provider**	**28**
Gynecologist	3
General Practitioner	3
Midwife	19
Lab Scientist	1
Lab Technician	1
Health Technician	1
**Level of Health Authority**	**5**
National Ministry of Health	2
Regional Ministry of Health	2
MRIS	1
**Other**	**1**
Dean of Medical School (Gynecologist)	1

### Coding and analysis

The interview data were subjected to both deductive analysis, based on the four initial program theories, as well as inductive analysis, in which a fifth program theory was identified as it emerged in the coding process. First, JB and LN created a codebook based on the four initial program theories, with subcodes for each context, mechanism, and outcome. Then JB coded the 34 transcripts in Dedoose [26]. Early in the coding process, VPS, JB, and LN identified an additional program theory, defined the context, mechanism, and outcome for that program theory, and then JB re-coded the transcripts to account for all five program theories. Once the transcripts were coded, VPS, GM, JB, and LN used the codebook to conduct a manual content analysis of the interviews to refine our program theories and choose the quotes to include in this manuscript. We used the RAMESES II Reporting Standards for Realist Evaluations [27] to report our findings.

## Results

Most participants confirmed and refined Program Theories 1 through 3. Only certain participants (health authorities who handled data) confirmed Program Theory 4, and we identified Program Theory 5 regarding provider empowerment during the coding process. Program Theories 1 through 5, presented as context-mechanism-outcome configurations, are presented below in Fig 1) and individually, in italics, under each program theory’s respective subtitle.

Due to the numerous quotes for each context, mechanism, and outcome for each Program Theory, we have kept a few quotes in text and exemplify additional quotes in Table 3.

**Table 3:** Additional Quotes.

Program Theory 1	
Context: hierarchical health system	*The Ministry of Health... only disseminates orders. And it does not dedicate much to follow-up, or to how you are going to do it. It just says “do it….” And what’s worse is that it doesn’t give you sufficient resources to do it.—*Regional Hospital Gynecologist
	*When I went to some trainings [on patient counseling regarding TA], I saw that some doctors... did not like the idea that the midwife is seeing this [how to perform TA].—*I-2 Health Post Midwife
	*No matter how much the Ministry of Health makes norms, laws, it never brings anything good, they are just orders. They do not consider in the moment, “how is it going to work, what is wrong, why is it not happening, why are the midwives not advancing, what is happening, what are they missing?” No. They have never been concerned [about that].—*I-3 Health Center Midwife
	*This is where one understands the importance of women making decisions about their own health. In other words, the concept of women’s empowerment regarding their own care. This is something that has never been considered, the biggest problem that exists in all Latin American countries, in which a small group of specialists who are gynecologists, masters of whatever, but who think they can decide for women, without understanding the problems of a woman in a poor country on a daily basis. It’s very complicated.—*Former Ministry of Health National Cancer Director
	*Strengthening the primary level is something we talk about that I think is already a paradigm. We all know that primary care should be fundamental. It should be, but it isn’t, and it fails to be that. We see the messaging, we see the policy, we see the decision-making. The decision-makers keep saying, “I’m going to open more hospitals. I’m going to solve cancer by building 24 cancer institutes,” and that’s not the solution.—*Ministry of Health Cervical Cancer Technical Team
Mechanism: bilateral exchange of information and ideas and grounding of new SAT program with provider buy-in	*Like I said, the biggest lesson learned for me is mapping. Map, map, map, because if you don’t know how to map, it will be very difficult for you to make any change.—*Former Ministry of Health National Cancer Director
	*Here, the people in charge are very involved, and that is something nice. Because for us as health personnel, they make us feel that we are not abandoned, that we are not like, “take this and go, and you see what things [are needed],” you know? Here it’s not like that. Here they guide us, they ask for our opinions to be able to improve. It is a nice thing.—*I-2 Health Post Midwife
	*By having constant trainings with the project, when we met, all the professionals were there, not only the midwives, but everyone involved in the project.—*I-2 Health Post Midwife
	*We are already seeing this change... the doctors are recognizing that midwives, in one way or another, are also a source of support for the doctors.... It’s become clearer that they complement each other. Each professional has their own knowledge and experience.—*I-3 Health Center Midwife
	*I remember [the planning workshops]. You divided the people from the [local] hospital by their job functions. This is what I call openness. For me, openness was important, and you made us all participate. I think that the outcome of that participation, of the entire event, was the openness. The more you open up, the more issues you can consider, but if you are prepared, you can solve them.—*Regional Hospital Laboratory Scientist
	*It was very clear to me that the partnership was so important. Even though in this case I am part of the Ministry of Health, I cannot go alone and make these decisions. I believe, even if it is in another program, even if it is in another place, I will always look for those other voices to nurture the decisions that are going to be made.—*Ministry of Health Cervical Cancer Technical Team
	*The goals now are not only screening, but also how many women have been treated and received follow-up.—*I-3 Health Center Midwife
	*The continuum of care, I think, is clear. You take the sample, deliver the result, and perform timely treatment. Then, the patient is happy. Many of the patients don’t return, or get lost on the way, because of the delay in getting results, delivering results.—*I-2 Health Post Midwife
	*You considered our opinions, our suggestions. And I liked that. In other words, you have taken every word, every experience that you have had in each site. This was not done overnight. It has been a very succinct project, a very organized project, evaluating each process that was occurring. It has not been easy because I remember that we had meetings until late at night.—*Local Hospital Midwife
	*Not judging, not treating anyone as beneath you because you are not part of my team or my group, because we are all part of this. We are all going to build something, so we have to create this equitable, egalitarian space and try to build from there.—*Ministry of Health Cervical Cancer Technical Team
	*First, I have to standardize the care, that is, learn the current continuum of care and understand it. Learn which part I can modify. Of course, some steps will be more complicated to modify because they concern structural issues... This identification of the problem that is still latent is important. That was an important lesson, the process mapping, and identifying the pending issues, and how they will help me.—*Former Ministry of Health National Cancer Director
Outcome: health providers’ ownership and recognition of the importance of the new SAT program	*First, you designed the strategy from the beginning, with a starting point, which was [the Micro Network], it was a pilot. Then, you added the policy component... because the policy decisions will help women get care at a national level, not only the small group that was in the pilot here.—*Local Hospital Midwife
	*I think that yes, those of us who have been there, who have been advancing along the way, we have seen that it is an achievement to have this type of intervention, and especially the implementation that has been gradually taking place with the equipment, the materials, the professionals, a whole scheme to be able to provide care for and reach the population.*—Former MRIS Cancer Coordinator
	*Finally, people assimilated that this is the right thing to do, isn’t it? [This intervention] came from the people themselves. That’s what we were going to do, right, and that was the common sense…. What should I do to be able to have a greater impact, with the services I provide. And that is how we should continue to operate.*—Regional Hospital Gynecologist
**Program Theory 2**	
Context: prior cervical cancer prevention program’s high level of patients lost to follow-up	*We don’t refer the patients [who are HPV positive] to the hospital anymore, because in the hospital they would get lost, the patients would get lost and they would not come back.—*I-2 Health Post Midwife
	*Because the patients rarely went to the hospital [for follow-up] and they were lost. They would go to the Regional [Hospital], then they would also go for treatment, but in the end, they were also lost and from there we would see what we could do, what our treatment metrics were like.* —Former MRIS Cancer Coordinator
	*The results are almost immediate now. It is not like before, it took six months, even a year. Sometimes they disappeared.*—Regional Ministry of Health Authority
Mechanism: task-shifting screening to women via self-sampling and task-shifting treatment to the primary level	*When a patient is positive with the [HPV] molecular test, we do her VAT, and sometimes she comes out positive, but it is not occupying the full 70% [of the cervix], and we treat her there [the primary level]. We no longer refer her out. But she does get the treatment. This also reduces patient referral and patient loss, and we treat her timely. That is why I say that the follow-up is reduced.*—I-4 Health Center Technician
	*Every patient of mine who tests positive, I send her [to the health center] and it is easier for her to go to a small health center to receive treatment before going to a hospital. You know that in the hospital it’s so cumbersome in terms of paperwork, referrals, queues, making appointments, and all that. So, that is one of the things that my patients like, that they are seen very quickly, and in a very cordial manner there at [the health center].*—I-2 Health Post Midwife
	*That has been one of the best decisions that I think has been made in the Ministry and managed to be implemented.... One was to try to do molecular testing, to even try to do it. And the second thing was to migrate the management of premalignant lesions to the primary level of care. That implicates what you said, strengthening the primary level through trainings, etc. These two things are what have worked best from my perspective, and what really caused change*.—Ministry of Health Cervical Cancer Technical Team
	*We have seen that this is a simple, practical, quick and easy treatment.—*I-4 Health Center Doctor
	*Now there is close coordination with the regional hospital, with the oncology center, with a committed [specific] person... Now there is an exact referral and coordination with the oncologists.—*Local Hospital Midwife
Outcome: higher rates of completion of the continuum of care	*We have been congratulated by the Ministry of Health because we have improved tremendously. We are increasing our coverage and our care. And really, it gives us a lot of pleasure, a lot of joy.—*Regional Hospital Midwife
	*It is professionally satisfying to be able to provide timely treatment to these women*.—Progreso Health Center Midwife
	*And there is a larger population that comes for this screening, and above all the treatment that is timely; that is, to have the thermal ablation at that same visit and to do it to the woman, and this makes the women with positive results seek us out, because of our commitment to treat them, and that is beneficial, above all, for the population.* —Former MRIS Cancer Coordinator
**Program Theory 3**	
Context: fragmented monitoring and evaluation systems for the national cervical cancer prevention program	*We had been managing our system based on patterns in the health records, but they did not help you find the positives and negatives in an agile way.—*Regional Health Authority
	*One of the biggest time wasters we have in our care is the amount of record keeping we have to do.—*I-2 Health Post Doctor
	*Many were lost because they were not working with a follow-up registry... in the peri-urban and rural areas, most of them were not taking this into account. It is their weakness. So basically we had to work more on follow-up records so as to not lose our patients.*—I-1 Health Post Midwife
Mechanism: implementation of a new data system that tracks the patient through the continuum of care	*So, we do our own record-keeping here because we saw what happened when we used to send [patient data] to statistics, they would tell us “there is no one to analyze it, nobody has the code,” and it would stop there.—*Regional Hospital Midwife
	*I think from the beginning I had this idea that we should not start without an information system. You reinforced that... sometimes we need that push of “Ok, you are on the right track” to be able to continue. Because the truth is that there are a lot of ideas, but putting them into operation is a different matter.—*Ministry of Health Cervical Cancer Technical Team
	*If there is better follow-up and awareness, we can have adequate results. If I, as a professional, give follow-up, and also the Regional Ministry of Health shows interest in the follow-up through the data registry, through the system, then it will allow us to work in a harmonious way and be able to handle follow-up.*—I-2 Health Post Midwife
Outcome: improved patient tracking	*The loss to follow-up of those who have not been treated has improved a little bit, because for Pap smears, that gap was large. There were many women who tested positive and the result was not delivered and then a year went by, the result was still not collected. And the HPV test alleviates that, and its implementation has helped a lot.—* Former MRIS Cancer Coordinator
	*In the [public health system], we have many problems because not every health facility in the MRIS has internet. All the I-3 and I-4 facilities, the regional hospital, they always have internet, that is, the [larger health facilities] have it, but not at all times. And the system, when there is a system in the cloud where you are entering [data], when there is internet, it is updated. So, at any moment, an I-4 facility can implement it. The smaller health facilities would have to send their information to be entered.* —Former MRIS Cancer Coordinator
	*In the creation of the forms for collecting patient information, there were data, aspects that were not relevant, that did not help streamline the process.... And in the end [the midwives] also realized that it took up more time, and so on. And maybe we should not have tried to unify a single information system. But at that time we didn’t have it. We had to start from something and not simply continue the old system.—*Regional Health Authority
**Program Theory 4**	
Context: fragile, under-resourced health system in a politically unstable country with frequent regime changes	*Sometimes health authorities promote doctors to be program directors. As I mentioned earlier, one doctor had already been trained [in TA] and then he was transferred to another facility, so there we are losing human resources. Human resources already trained, because you spend money, you spend time, and then to transfer them to another facility, and they are not going to perform the treatment, it is also a loss.*—I-1 Health Post Midwife
	*Of course, we ask the Regional Ministry of Health for human resources, but they always tell us that there is no budget, and in the case of the cancer program, we have almost no budget. There is a greater budget for nursing, vaccination, even for maternal care, but very little arrives for cancer, because they are almost forgotten programs, there is no budget for our area.*—I-2 Health Post Midwife
	*Finding people to screen is not the problem. The problem is when the patient comes, and reality hits. What are the realities? Realities like the state of our hospital. And if you’ve worked here a long time, you see that it doesn’t change. I have worked in the public health sector here for 31 years. The problem is political.*—Regional Hospital Midwife
Mechanism: empowerment of health care champions and continuous engagement between health providers and the research team	*The doctors have been sensitized, even those who are not gynecologists, who have wanted to help and work for the population, especially for women. So that has been very important for them to also contribute, and not expect something more from the project, because many times the professional says “how much are they going to pay me? What are they going to give me?” I haven’t seen those types of interests in this project because there have been sensitized people. That is the word, sensitized, so that they can support women’s health.—*Local Hospital Midwife
	*The midwife [name withheld]... is a very concerned, very responsible midwife... And the most important thing, she takes care that the patient does not drop out of care because of any delay. A delay during care, in the triage part or during treatment, or if the patient is referred to the specialist... She has been the contact who has made it possible for all these professionals to come [to the rural area] so that the patients can receive the treatment here. As I said, it depends on each patient, their availability, their financial resources.... We always help the patient financially so that they can come from far away or if they cannot come, then we also go look for them, using our own financial means. What we are most interested in is to achieve the goal that this patient be diagnosed, treated, and able to say, “I have been cured.”—*I-2 Health Post Midwife
	*The partnership, which has to do with teamwork. We started this project almost with me not even being a team leader. I met you when you came to the Ministry with other people. And well, I gave my opinions there, but very underground. And now I think it has been changing over time, as you said, but that relationship has been maintained. And that complicity is necessary to a certain extent. “Ok, now it’s your turn.” Like in relationships, right? Now it’s your turn to put in a little bit more, because I’m not going to be able to do it. So, that’s what it’s all about, the balance.—*Ministry of Health Cervical Cancer Technical Team
	*It hasn’t been like, “take this, here’s how this is done, here’s how that is done” and they leave you, right? No. You’re still here! There has been a permanent and lasting accompaniment over time, because we have to keep improving and improving and improving.—*Regional Ministry of Health Authority
	*I think that one of the most important things is this back-and-forth relationship with [the research team]. It has allowed us to exchange opinions many times, which helped us make final decisions... what we need is this kind of support, and you provide that through scientific evidence, sometimes by providing us with materials that you yourselves prepared. So that helps us a lot. Or your experience, because you have also seen other pilots. So all this nourishes us so that the program can improve and so that we can make the right decisions.—*Ministry of Health Cervical Cancer Technical Team
Outcome: program sustainability	*But we as a staff... are committed and we continue... whoever comes in, we continue with the work.—*I-4 Health Center Midwife
	*So, now with the continuity, and with this new norm that has been applied, the intervention has been improving, it has been resuming. But equally, the commitment of each professional, starting from the Ministry of Health, from the Regional Ministry of Health, from the organizations, has helped to maintain and continue this project.*—I-2 Health Post Midwife
	*The successes that were obtained in this program, in the development of the program, were transformed into policies! Into public policies that in one way or another would benefit not only the people of Iquitos, even though it was developed here in Iquitos.*—Dean of Medical School
**Program Theory 5**	
Context: limited quality continuing medical education and training	*The Ministry of Health regulates it, it sends you the clinical guideline and says, “This is the new way the new method will be applied.” Sometimes there are trainings with small groups, sometimes not, sometimes they just tell you to apply it, there are the instructions, and go.—*Regional Ministry of Health Authority
	*To improve we have to train more personnel, I am talking about our health system as a whole.*—I-4 Health Center Doctor
	*But when you ask the midwives how many have been trained, very few, and the vast majority that do VIA they are not well trained in diagnosis.*—Regional Hospital Gynecologist
Mechanism: dynamic trainings and program materials	*The strength has been that the primary level personnel have been trained, they learned the work and the mechanics, all the methodology. And they have been the ones who have strengthened [the program], who have made us move forward.—*Former MRIS Cancer Coordinator
	*The way the facilitators have contributed has been very, very good. Because they have sensitized all the health personnel... It has been the way they reached the midwives. And [the midwives] are still motivated to this day. They themselves are asking for the results. So, so, so, so available.—*Regional Ministry of Health Authority
	*We have been working and contributing ideas on what the information sheets or cards should look like... we have worked collaboratively, giving ideas so that the information could be accessible to the population.—*I-2 Health Post Midwife
	*Or “What if I don’t take the sample correctly?” We had to explain it to them, and to do that, we used the flip charts and the images a lot. That helped us a ton.—*I-2 Health Post Midwife
	*The flipcharts that were created were their own little study, their sensitization. Because they were really from the people and for the people.—*Local Hospital Midwife
	*More than anything else, the practice sessions, the dynamic nature and all that... because theory, well, you know... What wakes you up are the dynamics, the practice sessions. That is why here they are doing almost 100% practice sessions in these workshops we are having. So they are not boring.—*I-3 Health Center Midwife
Outcome: empowered health care providers committed to the program’s success	*And the way of reaching the woman, and the acceptability [of the message] according to the counseling, was important when we gave the result, without scaring her, breaking her preconceived notions... and they accepted the treatment, we have had almost no rejection. In other words, there has been a small percentage, but hardly any compared to what has been achieved.—*I-2 Health Post Midwife
	*But there are professionals who do like the subject, they get more into it. I think that Dr. [name withheld] is still there in [an I-2 health post]. Dr. [name withheld] is trained, and Dr. [name withheld], but the one who has given the most has been Dr. [name withheld], he is still in [an I-2 health post]. He is not leaving [that health post].—*Former MRIS Cancer Coordinator
	*There are colleagues who have not done the training, they are new and we, or at least I, who have been trained, guide them on how to do it. So that is the sequence.—*I-2 Health Post Midwife
	*It feels great to see how [health professionals] are taking these initiatives for the benefit of their own population and empowering themselves by asking for more resources. They already have their own ideas, so it is very gratifying to see these changes.—*Ministry of Health Cervical Cancer Technical Team

### Program theory 1

*In the context of a hierarchical health system, the bilateral exchange of information and ideas and the grounding of new SAT program with provider buy-in led to the health providers’ ownership and recognition of the importance of the new SAT program.*

#### Program theory 1 context.

Participants agreed with our identification of a hierarchical health system as the relevant context. In fact, they identified four distinct hierarchies that comprised the hierarchical health system: the hierarchy within health professional roles, the Ministry of Health’s structural hierarchy, the hierarchy of the public health facility system in the MRIS, and global hierarchies based on geography (Table 4).

**Table 4:** Hierarchies Identified by Participants (Highest to Lowest).

Professions Hierarchy	Ministry of Health Hierarchy	MRIS Health Facility Hierarchy	Global Hierarchy
Onco-Gynecologist	Ministry of Health Headquarters - Lima	Regional Hospital (Tertiary Level)	Global North
General Practitioner	Regional Ministry of Health (GERESA)	Local Hospital (Secondary Level)	Global South – Capital/Urban Areas
Midwife	MRIS Leadership	Health Facilities (Primary Level, I-4 to I-1)	Global South – Regions Outside Capital/Urban Areas
Health Technician			

For example, participants attributed some health providers’ and authorities’ initial resistance to the intervention’s task-shifting of treatment from the hospital to the primary level of care to the medical professional hierarchy.

*In the Ministry of Health’s system, before the [intervention], it was unthinkable that the primary level could do this task [provide treatment]. Unthinkable! You would say that the patient has to see the specialist!—*Regional Hospital Gynecologist

Others identified the Ministry of Health’s hierarchical culture, with its top-down structure and emphasis on numerical targets, as an impediment to patient care or as an inaccurate depiction of how patient care is actually carried out on the ground in remote areas outside the capital:

*It has always been a weakness for us in the system that we cannot work together in an integrated manner, because the staff has functions and goals that they have been instructed to meet; for example, the nurses are very focused on meeting their indicators... and perhaps there is a lack of good organizational capacity to be able work together.—*I-4 Health Center Doctor

Finally, others identified the hierarchical nature of global public health, where so-called “experts” from the capital cities or Global North impose their solutions without bothering to understand the reality on the ground:

*One of the things that differentiates PPC from other projects is that other projects want to impose a conceptual model. On one hand, this is fine because you are testing it. But they want to put their processes in place; that is, they want to do things their way without first understanding the reality of each geographical area, of each regional government or local government. And that is the key point, that is where it fails.—*Former Ministry of Health National Cancer Director

Participants reported that these various hierarchies made those on the ground, at the lowest levels of power, feel abandoned when it was time to put new clinical guidelines or models of care into practice: they receive orders that must be operationalized, without the resources or time to make them happen.

#### Program theory 1 mechanism.

Participants agreed that our hypothesized mechanism of bilateral exchange of knowledge and ideas and grounding the program with provider buy-in reflected their experience. They identified three examples: (1) shared mental models, (2) consideration of multiple perspectives to solve the problem, and (3) pre-implementation process mapping and step-by-step planning.

**Fig 2:**
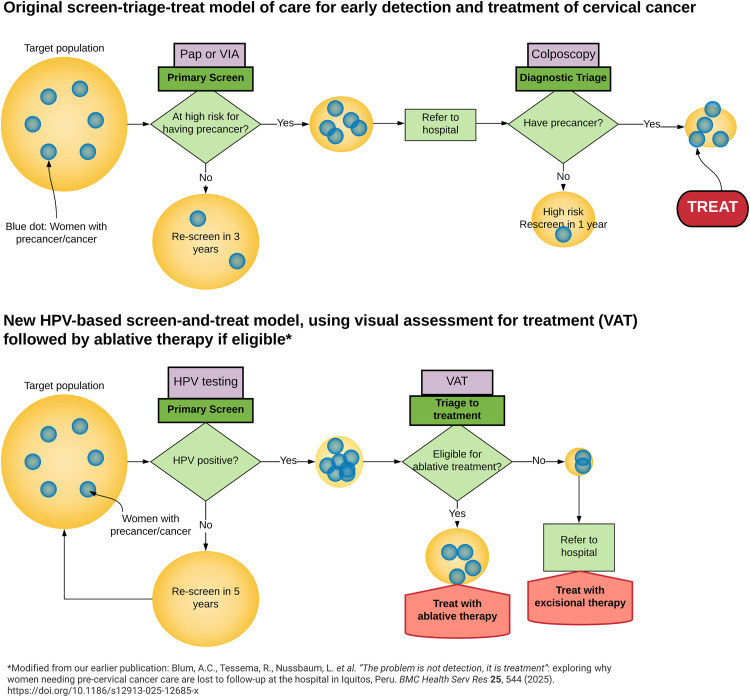
PPC Mental Model.

PPC introduced two different mental models to local stakeholders as part of its implementation strategy. First, the screen-diagnose-treat mental model demonstrates how not just screening, but actually completing the continuum of care, is crucial to cervical cancer prevention efforts (Fig 2).

**Fig 3:**
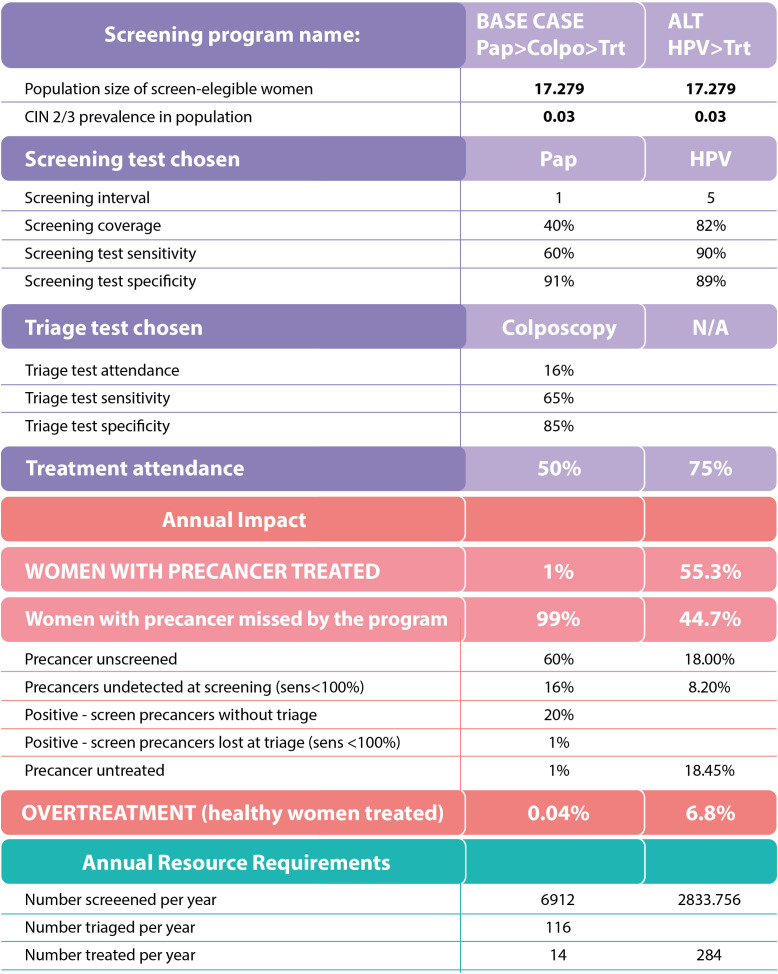
PPC Scenario Analysis Tool.

Second, the cervical cancer screening scenario simulator tool shows how decisions made at one part of the health care system impact another part of the system. In other words, the scenario simulator tool allows stakeholders to test “what if” service delivery and resourcing strategies, with the goal of identifying options with the highest likelihood of working under realistic assumptions about possible outcomes (Fig 3).

*Those slides [regarding the continuum of care] were fundamental. Those mental pictures gave us the practical understanding and the schemas for the continuum of care, and this was very important for decision-makers. From those you can understand how the processes occur. The other tool that struck me was the [scenario analysis] tool. When you presented the loss percentages, it was brutal. I mean, it was very clear, very concise.* Former Ministry of Health National Cancer Director

The stakeholders’ adoption of the shared mental model that treatment was just as important as screening for cervical cancer prevention was a crucial mechanism for the program’s success. Participants emphasized that the shared understanding of the importance of follow-up care was crucial for them to shift their mindset from only focusing on screening target numbers to also following up with those who screened positive to get them into treatment.

*That was what we worked on a lot; that is, we had established goals of screening, screening, screening, and we managed to screen a percentage. We were fine, but nobody stopped to think or reflect,“hey, and these positive results, what about these women?” Then we began to create other goals, not only about screening, but also about delivering the result, and then came, “hey, don’t just deliver [the result], treat, treat, because then you are culminating this whole process of prevention.” Then... if she merits attention at another level [of care], that’s something else, but you, at the primary level, you got as far as you could, you exhausted everything you could do. And there were a number of other indicators that we added so that we could have women whose cancer was prevented.—*Regional Ministry of Health Authority

Participants also reported that the project’s methods of centering the local perspective, creating a horizontal culture of mutual respect regardless of professional role, and meeting continuously to deeply understand the local situation on the ground were key to its success.

*I really liked it when [VPS] said, “Let’s look for where the bottleneck might be, what do you think? …You all are the ones in the field, how should we do this?” It was important to participate and give our point of view of the reality that we have here.—*I-2 Health Post Midwife

*The fact that we jointly built the continuum of care for these patients, which is grounded in our reality... working with the primary level, working with the secondary, tertiary levels and building the continuum of care together, was, I believe, what most facilitated a positive outcome.—*Regional Ministry of Health Authority

Finally, participants remarked that the slow, deliberate pace of the project, which incorporated theory and made time to clearly identify the health system’s obstacles and gaps, was new, and that this pre-implementation, step-by-step planning, rather than simply reacting to emergencies as they arose, was a critical mechanism for the project.

*The continuum of care, how the samples are going to be taken, who is going to receive them, the registration of the samples. The coding of the continuum of care. This whole system has been built, little by little, in a progressive and systematic way.—*I-2 Health Post Midwife

#### Program theory 1 outcome.

Participants agreed that the outcome of Program Theory 1 was the health providers’ ownership and recognition of the importance of the new SAT program.

*How do I do it? How do I implement it? How? It is step by step, that’s what we told ourselves, that’s how we started. But in the end we already have a goal, an objective, which is to treat, we already know how to do it. I’m not trying to say we are the best ever, but we keep going, don’t we? Finding our patients, seeing them, reducing cancer. That’s what it’s about.—*I-2 Health Post Midwife

### Program theory 2

*In the context of the prior cervical cancer prevention program’s high levels of patients lost to follow-up, task-shifting treatment to the primary level led to higher rates of completion of the continuum of care.*

#### Program theory 2 context.

Participants confirmed and reiterated PPC’s prior findings that Pap smear results were slow, and patients were lost along each step of the continuum of care, particularly between the screening and treatment steps, because treatment of abnormal Pap smears took place at the hospital, not at patients’ primary health care facilities [5,7].

*Before there was ablation, there was cryotherapy, which was more cumbersome, it took a long time…. And if you wanted to go to a rural area, you could not, because you had to carry the tank, and many times transportation would not take you, because the tank was so big. To move that and to move the professionals with the limited budget that existed did not allow the professionals to go and do the [treatment].—*Former MRIS Cancer Coordinator

#### Program theory 2 mechanism.

Participants agreed that task-shifting screening to a superior tool (HPV testing) that also allowed women to self-sample and task-shifting treatment (with VAT and TA) to the primary level reduced barriers and improved the care experience for both patients and providers.

*The practicality of the [HPV] test. This test can be a self-test, which helps the woman to perform it during a home visit, in a campaign. Patients widely reject other preventive cancer tests due to fear, or stigma, or the embarrassment of being seen. So this test has allowed us to break down that barrier that we faced as health professionals.—*I-2 Health Post Midwife

Participants also noted that their idea of making thermal ablation treatment portable reduced barriers to treatment.

*That is the advantage of this treatment, that it is something that you can move around, it is so practical, you can go anywhere, you just take your little case with your thermocoagulator, your probes, and that’s it.—*I-4 Health Center Doctor

Participants agreed that minimizing referrals helped retain patients and get them into treatment.

*The treatment is available at primary level sites... it’s close to our health center, we can take the patients there. I have taken many of them. Sometimes I say “Ma’am, I’ll take you on my motorcycle!”—*I-3 Health Center Midwife

*It is really satisfying because I know this population, many of their mothers had passed away from cancer. They were crying. And they themselves were saying... “we should have had this [treatment] before,” for their mothers’ benefit. And that is why they came on their own, and they told all their neighbors to get tested, because the treatment they had seen was very easy!—*Regional Ministry of Health Authority

#### Program theory 2 outcome.

As a result of using superior screening and treatment tools, particularly the self-sampling option and task-shifting treatment to the primary level, more women completed the continuum of care.

*People already know about [the intervention], it is already well known, people are coming to ask for the [HPV] test. Even patients who do not have public health insurance, who have employment-based insurance, they come from different places because they already know about the efficiency of the results, about the efficiency of the treatment.—*I-2 Health Post Midwife

*The providers’ commitment and the continuum of care have made it possible for us to break down these barriers and for patients to be able to come to a health facility... they are aware that we are doing a swab test, they ask for it. That means that the message is reaching more and more women. We are empowering, we are educating, and a patient who is well cared for, a patient who has received a diagnosis, whether positive or negative, and the continuum of care has been fast, that service that you have provided has been adequate—that patient will come back, and it will have a multiplier effect with her cousin, with her sister, with her neighbor, with her aunt.—*I-2 Health Post Midwife

To further improve the intervention, participants suggested procuring more thermocoagulators so that treatment could be provided at more primary level health facilities, and solving ongoing supply chain delays associated with the HPV testing materials that resulted in some delay of results, which in turn caused more delays along the continuum of care.

### Program theory 3

*In the context of fragmented monitoring and evaluation systems for the national cervical cancer prevention program, implementation of a new data system that tracks the patient through the continuum of care improved patient tracking.*

#### Program theory 3 context.

Participants confirmed and reiterated the project’s findings regarding fragmented, overlapping, and not user-friendly health records systems that were both electronic and paper-based (manuscript in development). They noted that there was a lack of standardized treatment codes, that the reference/counter-reference system between the different levels of care was slow and often non-functional, and that all these problems were exacerbated by unreliable and/or a complete lack of Internet access, depending on physical location and the health facility’s level of care.

*We keep repeating everything manually. If we had an integrated networked system, maybe it would be easier to do patient follow-up... What we need to do is to systematize this, we keep doing the registering. One of the biggest time wasters we have in our system is the amount of record-keeping we have to do.... If we have really been able to reduce this loss to follow-up, it has been because you [the project] have been behind it.—*I-2 Health Post Doctor

#### Program theory 3 mechanism.

To monitor and evaluate program reach and impact, the research team built a hybrid paper/electronic screening registry called SIMOPP (Sistema de Monitoreo del Proyecto Precancer). In collaboration with selected health professionals and authorities, we (VPS, PG) co-developed new clinical forms tailored to midwives’ needs. These triplicate forms ensured that midwives retained a copy, another accompanied the cervical sample to the lab, and the third was collected by the research team for electronic entry. Midwives delivered cervical samples to the lab located at a primary level facility—ranging from daily to every two weeks, depending on distance—and retrieved patient results. Patients with negative results required no further screening for five years, while those with positive results were promptly scheduled for VAT at a primary level facility and, depending on the results of the VAT, either TA treatment during that same visit or referral to a hospital.

However, follow-up for hospital referrals was challenging, as midwives lacked direct updates unless they personally contacted the hospital midwife. To address this gap, a PPC research assistant used SIMOPP to track women referred to the hospital level and verify care resolution. Specifically, a PPC research assistant (a nurse) searched the hospitals’ electronic system and handwritten colposcopy logs for the names of referred women up to three months post-referral, allowing sufficient time for patients to access care; this information was uploaded into SIMOPP. The team printed monthly reports that included women who had not yet received follow-up care for the MRIS cancer coordinator, who then updated midwives on referral outcomes, enabling them to follow up with patients lost to hospital follow-up.

Those who were involved in the creation of SIMOPP and had access to the system recognized its value; i.e., that it permitted providers to see how their patients were doing without depending on an outside system.

*I think from the beginning I had this idea that we should not start without an information system. You reinforced that... sometimes we need that push of “Ok, you are on the right track.”—*Ministry of Health Cervical Cancer Technical Team

#### Program theory 3 outcome.

These participants recognized that SIMOPP facilitated improved patient tracking.

*By creating an information system…, this is the starting point, to visualize what I am doing. It gave me a good sign, that I am on the right track, because if I don’t have that and they tell me, where I am going today, I don’t know where I am going, what I am doing. Am I getting good results? Am I getting bad results? You could lose track of time, you could even get discouraged. Well-integrated information systems help us measure ourselves.—*Regional Health Authority

However, these participants were in the minority. Most participants expressed a lack of knowledge regarding the data system because many of them did not directly interact with it. Rather, they filled out their clinical form (entered manually into SIMOPP by someone in PPC), and then, monthly, they obtained a list of their patients requiring follow-up from the MRIS cancer coordinator (based on SIMOPP output), likely without information regarding where the data came from. For participants who worked in more rural areas, where there aren’t computers or stable internet access, this program theory was not proven for them.

*There should be a database connecting the hospital data with the primary level facility data... I would like to change the registry and the record-keeping to make it more didactic. The information network would be more practical... I have the [Ministry of Health] registry, it has some information, but we have to register other documents separately.—*I-4 Health Center Midwife

### Program theory 4

*In the context of a fragile, under-resourced health system in a politically unstable country with frequent regime changes, the empowerment of health care champions and continuous engagement between health care providers and the research team led to program sustainability.*

#### Program theory 4 context.

Participants agreed with our identification that frequent staff turnover and political regime change caused constant disruptions to health care delivery. They lamented that the health system often invests in staff trainings on a particular system process or clinical task that is specific to that staff’s role, then that staff member is assigned to a different region or position, and then the next person must be trained all over again, resulting in a loss of resources.

*Because there are just changes in government, changes in personnel, and each one comes with different ideas. Not everyone is committed to fighting cancer.—*Regional Hospital Midwife

#### Program theory 4 mechanism.

Participants agreed that project champions with intrinsic motivation inside the health system were a crucial mechanism.

*The pandemic is another thing we went through. The health facilities did not have normal hours for about three years. The clinics were closed, and with the introduction of the molecular test, you took advantage of that time to get all the health providers involved, trained, and participating, and they sent the patients [for treatment] when they found larger lesions. And that is where cancer is increasing. Cancer itself is not increasing, but they are discovering it early, and thank God we also have an oncology center at the moment, where we have an oncologist committed to this task and doing timely treatment.—*Local Hospital Midwife

*One of the great strengths of this facility has been Ms. [name withheld]... many people, for example, have had the wrong dates, patients coming from other facilities, she... took the time to explain to them, for example, there have been times when it was not the date we do treatment, because we have only one day to do TVT and the patient came, she looked for a way to have it done on that day, because we understand that... most of our population is low-income. So asking the patient to come back another day would be practically telling her that she won’t get treatment because the patient might not be able to come back.—*I-2 Health Post Doctor

The constant accompaniment of PPC staff was another key component: as external allies and champions, as liaisons between the primary and tertiary levels, as facilitators and motivators, and also liaisons between local professionals and international experts, allowing individuals to feel confident in system choices that they were making.

*The group of professionals who have been guiding us step by step, doing the follow-up, seeing our work from the bottom up, from the health facility itself, because in reality you have gone to all the health facilities to see how we are working, if we are doing it well. In other words, you have not just taught us and left us, you are always there behind us, supporting us.—*I-2 Health Post Midwife

*The person who is supporting us a lot is [a member of the PPC research team], she is the one who is giving it her all. If you have any setbacks, she tries to solve them so that you can continue. Her work is also praiseworthy because she is always involved.... We have had a little hiccup regarding the testing of the swabs. The swabs already had an expiration date, but the cartridges did not, so we had been told that we could only work for a month, because after that we wouldn’t be able to use them. But we continued considering the issue and thanks to her, she coordinated with an international expert... she is the support, she was already looking for international technical assistance…. And this international technical assistance has been important, of course, because you can be a mechanic, you can put in a sample, insert it and you get results, but the whole process has to be accompanied by a theoretical and scientific explanation, and we have really had that. We have had several visits here, several visits from experts, where in a small room many things were being done.—*I-4 Health Center Laboratory Scientist

#### Program theory 4 outcome.

Participants agreed that the internal champions and external allies resulted in the intervention’s sustainability.

*And it did work because... there can be trainers, but also the team that you train has to be involved in what they want to do... and it worked, it really worked.—*I-2 Health Post Midwife

*And truthfully, as healthcare workers, we have the satisfaction of feeling that we are useful and that we are doing something good, something productive, by treating these women and preventing many more cases in the future.—*I-4 Health Center Doctor

### Program theory 5

*In the context of limited quality continuing medical education, dynamic trainings and program materials led to empowered health care providers committed to the program’s success.*

#### Program theory 5 context.

Participants identified the lack of quality continuing medical education as a key context. They noted that because health providers do not receive sufficient training, providers do not feel comfortable performing new procedures or using new techniques. This problem is exacerbated for health providers located outside the city, because it costs more for them to attend trainings.

*To improve we would have to consider two aspects, first, training more personnel in our health system, and second, to avoid rotations, the human resources reassignments that occur, because those make us lose a valuable human resource that has been trained.—*I-4 Health Center Doctor

#### Program theory 5 mechanism.

Participants stated that PPC’s dynamic trainings were both useful and motivating. Specifically, they appreciated that, as part of their training on the HPV molecular test and the self-sampling option, the female providers tested themselves via self-sampling so that they would have firsthand experience to inform their patient counseling on the new procedure.

*We may have the ability to reach the patient, but there were no techniques, with you we learned the techniques of how to be more understandable, you could say, so that the patient can understand you in a way that is basically saving her life, her health, in a way. You have taught us step by step with posters, with samples.—*I-2 Health Post Midwife

Participants also identified PPC’s high-quality materials for patient education as vital to the project, and appreciated their role in contributing to and validating the materials to ensure their local appropriateness and acceptability.

*It’s a nice thing when you suggest something, and it’s taken into account. And that flip chart has really helped us explain the self-sampling to our patients.... It helped me a lot, the more images there are, the more it helps. After explaining it all to her, the woman wants to see the image... Without that in the counselors’ offices we are just talking, talking, but she doesn’t pay attention to you, she gets bored.—*I-1 Health Post Midwife

#### Program theory 5 outcome.

Participants posited that the outcome of dynamic trainings and high-quality materials was empowered providers committed to the program’s success.

*The consistency, the permanence, the insistence help empower the personnel... and the services have made this work in cancer more important. In other words, you can see how the personnel are more empowered.—*Former MRIS Cancer Coordinator

*But it feels really good when a patient, after having completed her treatment, comes back to you and says, “thank you.” It is something inexplicable. You cannot explain it because it is beautiful that you know, and that remains in your conscience, that because of you or other people, because of teamwork that you have done well, that that mother of a family can have a better life, a full life accompanying her children, her husband. It is really beautiful. I believe that has helped us a lot.—*I-2 Health Post Midwife

Participants encouraged PPC to continue offering trainings so that more (and new) providers are trained, including more training specifically on patient counseling, i.e., how to explain to patients that a positive result is not cancer, as well as further training on VAT.

*And continue training, because the more health facilities there are, and the more trained personnel they have, the more accessible it is going to be. And we will be able to fight with greater emphasis against everything this cancer is, preventing cancer.—*I-2 Health Post Doctor

## Discussion

PPC conducted this realist evaluation to learn what worked in our cervical cancer EDT pilot in Iquitos, in what circumstances, for whom, and why, to understand the underlying mechanisms leading to acceptable and sustained change, to inform how PPC tailors its assistance in other regions in Peru. This realist evaluation revealed that continuous engagement with public health system providers and authorities in a horizontal manner across the system’s professional hierarchies (Program Theories 1, 4, and 5) and facilitating co-design and implementation of an HPV testing and treatment continuum of care that is patient centered and reduces patient loss to follow-up (Theories 2 and 3) can, ultimately, lead to improved screening and treatment rates.

PPC prioritized sustainability by deciding early on to work within the established health system, first in the MRIS, and then in collaboration with the national Ministry of Health once it adopted the HPV-based screen-and-treat approach we piloted in Iquitos. We did not want to create a parallel system; rather, we wanted to establish buy-in within the public health system and work with system actors to improve the system. Using an embedded implementation research approach [28] required us to be patient and progress more slowly. It took time to identify actors across all system levels and build their trust in our team and in each other so that we could work in partnership. But the results show that this was necessary; participants positively compared PPC to other interventions that simply import their established program theories into new settings (see global hierarchies section of Program Theory 1 Context). Because program stakeholders in Iquitos self-identify as partners and co-owners of this intervention (see Program Theory 1 Outcome), PPC has been able to shift its resources, slowly reducing its presence on the ground in the MRIS, without sacrificing the intervention’s sustainability because it is already integrated into the existing health system (see Program Theory 4 Outcome).

Different aspects of the intervention spoke to different providers. For example, few participants apart from the Former Ministry of Health National Cancer Director mentioned the scenario analysis theoretical model as a crucial mechanism (see Program Theory 1 Mechanism). For this participant, this tool was a critical aspect of the intervention, which is logical given this participant’s prominent policy-making position. The midwives, on the other hand, focused more on their feelings of validation and empowerment when the research team sought their input on educational materials (see Program Theory 5 Mechanism). Therefore, a complex intervention that impacts multiple levels of the health care system will ideally contain various components that incorporate different system actors and leverage their diverse levels of expertise.

According to the participants, this intervention also centered the patient (see Program Theory 2 Mechanism). This opinion is consistent with the results from a prior PPC study regarding HPV-positive patients’ perspectives on the acceptability and feasibility of the intervention, in which the participants expressed satisfaction with how quickly they received their screening results and were offered treatment, and hoped that the new strategy would be expanded to reach more women [29]. Replacing the former Pap smear- and VIA-based approach with an HPV test is patient-centered due to the self-sampling option, which has been shown in other studies and in the PPC intervention (manuscript forthcoming) to increase screening participation rates among under-screened populations [30] and prioritize patient privacy and comfort [31].

Regarding treatment, shifting treatment from the hospital to the primary level brought treatment closer to the patients so that they did not have to learn how to navigate accessing treatment in the hospitals, which come with their own idiosyncratic bureaucratic obstacles. Further, the intervention eliminated at least one step in the continuum of care for most HPV positive patients who were eligible for TA because they could receive it on the same day as their VAT. These results confirm TA’s advantages over prior cervical cancer treatment techniques, including reduced treatment time and increased portability [32]. The participants in this study overwhelmingly attributed increased patient screening and treatment rates (see Program Theory 2 Outcome) to these attributes of the intervention. However, patients continue to be lost to follow up even after this intervention was implemented. Persistently, women who live in more rural areas face additional barriers to screening [33] and treatment access [10,13].

Program Theory 3—that our new data system would improve patient tracking—was not proven for most participants. In other words, our data registry system improved patient tracking for the study team for monitoring and evaluating the intervention and for the regional cancer coordinator, but for most health professionals, it was simply a different clinical form they used, albeit one they co-designed. As HPV- and TA-based SAT is now scaling up throughout the country, there is a new Ministry of Health cervical cancer data registry system that health professionals who have electronic access are using on a more widespread basis. Nonetheless, the seemingly intractable barriers of consistent internet access and scarce IT resources remain. So long as these barriers exist, the health system as a whole will face challenges with making patient data systems useful for providers.

Returning to sustainability, while our trainings were a key mechanism to educating and empowering health care providers to counsel their patients on HPV screening and treatment, we plan to embed them into the Ministry of Health system, engage continuing medical education programs at the Colegio Médico del Perú (equivalent to the American Medical Association), and develop syllabi for teaching these topics at medical and other health professions schools. PPC is currently assisting the Ministry of Health in hosting trainings regarding self-sampling and thermal ablation, and has distributed its training materials to the regional cancer coordinators across Peru to disseminate to their health personnel. We have also added a section on HPV screening and treatment of precancerous lesions via TA to the Gynecology and Obstetrics course, the Scientific Research Methodology course, and the Community Medicine course at the sole public medical school in Loreto, for piloting and, hopefully, future scale-up to other educational institutions across Peru. PPC has influenced health system changes by relying on our history of open communication and respectful collaboration with key stakeholders in the health system; we will continue to work with key stakeholders to develop, pilot, and scale up training of current and future health professionals on the new screen-and-treat approach.

There are various limitations to note. First, much of the realist evaluation literature, including the materials for a realist evaluation course that VPS, SG took before embarking on this process, focuses on single component interventions, whereas this intervention had multiple layers and components. For example, this intervention changed the screening test and the treatment protocol, shifted follow-up care to the primary level, condensed the continuum of care, and created a new hybrid electronic/paper registry, all of which required the consensus of multiple stakeholders across system levels. Developing the program theories took more than one year as we tried to tackle the complexity of this intervention and reflect it accurately within the confines of the realist evaluation framework, a limitation that has been noted before [34]. Realistically, we are not sure we would use this framework again for a project with this level of complexity in a health system that is constantly changing [35].

Moreover, the realist evaluation framework was created in the Global North and does not consider the covert and overt power disparities that influence how candidly stakeholders will respond to leading questions that the realist evaluation requires [36,37]. We felt somewhat bound by this approach, where participants had to agree or disagree with the program theory statements we supplied. To address this limitation, as mentioned above in the Methods section, the research team conducted an initial focus group with 14 health care providers who were key local collaborators in implementing the intervention to inform the development of the four initial program theories. Then we began our interviews by asking open-ended questions about participants’ own reflections regarding which elements of the intervention were most helpful and which could be improved, so that respondents had the opportunity to provide their own input regarding the program theories without being driven by our initial theories.

## Conclusion

This realist evaluation showed that accompanying health providers through continuous engagement in a horizontal manner across the public health system’s various hierarchies (Program Theories 1, 4, and 5) and centering patients by facilitating an HPV testing and treatment continuum of care that is more convenient for them and reduces patient loss to follow-up (Theories 2 and 3) can ultimately lead to improved screening and treatment rates. Furthermore, we learned that where we embedded and partnered with the public health system to co-design and assist in implementing the transition from a Pap smear- and VIA-based screening program to an HPV- and TA-based SAT program, actions were sustained. These two aspects were sustained: both HPV testing (including self-sampling) and TA are now part of the national health policy for cervical cancer EDT, and PPC is providing technical assistance to the Ministry of Health to implement this new policy across Peru by facilitating knowledge exchange through trainings and material dissemination. These insights will inform our work as this project adapts and scales up in other parts of Peru.

## Supporting information

S1 Text Interview Guide (Spanish). (PDF)

S2 Text Interview Guide (English). (PDF)

S3 Text Abstract (Spanish). (PDF)

S1 Checklist PLOS Questionnaire on Inclusivity in Global Research. (DOCX)
