# Supplementary material for: A realist evaluation of a novel cervical cancer prevention strategy in Iquitos, Peru
Source: PLOS Glob Public Health. 2025 Nov 19;5(11):e0004517. doi: 10.1371/journal.pgph.0004517 (PMC12629488; doi:10.1371/journal.pgph.0004517)
Supplement: S1 Text — (PDF) [file pgph.0004517.s001.pdf]

“Desarrollando y mejorando estrategias para la prevención del cáncer de cuello uterino en Perú”

Guía para entrevistas para evaluación post- implementación con colaboradores

Al inicio de cada reunión de trabajo los participantes darán su consentimiento informado. En caso las actividades se realicen de forma presencial, el consentimiento informado será escrito, y en caso sea virtual o por teléfono, el consentimiento será virtual y grabado

I. Introducción

a. *Presentación y explicación de los objetivos de la reunión/ entrevista.*

Les agradecemos de antemano por su tiempo y disposición. Como sabe, el objetivo de esta entrevista es entender un poco más sobre su perspectiva sobre el Proyecto Precáncer en su región, incluyendo los resultados del proyecto del proyecto, los retos que influyeron al proyecto, y sus ideas sobre cómo ocurrieron los cambios en el sistema de salud y quién fue afectado por estos cambios.
No hay preguntas ni respuestas correctas o incorrectas- solo nos interesa conocer sobre sus experiencias y opiniones.
[Notar el rol y nivel de autoridad de la persona entrevistada: en el sistema de salud. Por ejemplo: “Obstetra. EESS”]

II. Preguntas

A. Logros

1. ¿Desde tu perspectiva qué piensas que han sido los **logros más importantes** de este programa de tamizaje y manejo de VPH?
    a. ¿Me puedes dar un ejemplo?
    b. ¿Cómo han sido estos resultados diferentes a los de otros proyectos (si es que lo han sido)?

B. Procesos

2. ¿Qué fue **diferente sobre este proceso** o manera de trabajar?
3. ¿Cómo ha sido este proceso diferente al proceso de otros proyectos?
    a. ¿Otras organizaciones
    b. ¿Otras iniciativas del sistema de salud?

4. ¿Piensas que hay otros que tendrían una opinión diferente sobre el logro o proceso más importante? ¿Por qué?
5. A lo largo del proyecto se realizaron diversas actividades y se usaron herramientas en cada parte del proceso **(LÁMINA 1: actividades y herramientas).**
    a. ¿Qué fue útil/ no útil sobre estos procesos?
    b. ¿Algo te llamó la atención sobre estos procesos?
    c. ¿Qué te pareció diferente?

6. ¿Qué lecciones aprendidas tomarías sobre esa experiencia para aplicarlas a otro programa?
    a. ¿Por qué eso es tan importante replicar?
    b. ¿En qué manera mejoraría ese proceso?

C. Teorías sobre procesos fundamentales

Pensamos que hay 4 procesos que fueron realmente importantes en asegurar los logros de este programa y nos gustaría saber tu opinión sobre nuestras teorías. Si no estás de acuerdo con nuestras teorías eso está bien: la información que nos estás dando nos servirá para formular teorías nuevas.

7. La primera idea que nosotros tenemos es que: **(LÁMINA 2: Teoría 1)**
    a. Primero quiero confirmar si estás de acuerdo que tenemos un sistema de salud jerárquico. ¿Estás de acuerdo? ¿Podrías dar un ejemplo?
    b. ¿Qué piensas sobre esta teoría?
    c. ¿Crees por ejemplo que entender la importancia del seguimiento influenció la decisión de tomar esta estrategia nueva de tamizaje?
    d. ¿Modificarías esta teoría de alguna manera?

8. La segunda teoría que tenemos es que: **(LÁMINA 3: Teoría 2)**
    e. Primero ¿qué opinas sobre la idea de que este sistema de salud, antes de la implementación de este programa, tenía un alto índice de pérdida de seguimiento y dificultades en la colección de datos?

    a. ¿Qué piensas sobre esta teoría?
    b. ¿Modificarías esta teoría de alguna manera?

9. La tercera teoría que tenemos es que: **(LÁMINA 4: Teoría 3)**
   a. Primero quisiéramos saber qué opinas sobre la idea de que los sistemas de M & E de datos son intensivos y frecuentemente desconectados de otros sistemas
   b. ¿Qué piensas sobre esta teoría?
   c. ¿Modificarías esta teoría de alguna manera?

10. La cuarta teoría que tenemos es que: **(LÁMINA 5: Teoría 4)**
    a. ¿Por último, en cuánto a contexto, estás de acuerdo que este sistema de salud ha tenido frecuentes impactos sistémicos como cambios políticos frecuentes o la pandemia de COVID-19?
    b. ¿Qué opinas sobre la teoría?
    c. ¿Modificarías esta teoría de alguna manera?

D. Resumen

En resumen, vamos a terminar con tres preguntas un poco más generales.

11. ¿Si pudieras cambiar cualquier cosa sobre este programa para que funcione mejor, qué cambiarías? ¿Por qué?
12. Mencionamos antes que este programa, o programas de salud similares podrían funcionar de modo diferente en lugares diferentes. ¿Qué tiene de especial este lugar que hace que funcione (o no funcione) este programa?
13. ¿Para terminar, qué más crees que necesitemos saber para realmente entender cómo ha funcionado este programa en tu región?

LÁMINA 1: Actividades y herramientas

• Talleres al comienzo del proyecto; primero solo obstetras, luego solo médicos, luego todos juntos	• Visualización de los detalles del sistema de salud y de la continuidad del cuidado de las mujeres en gráficos y el modelamiento del impacto de cada decisión
• Talleres de diseño para planear la implementación y sobrellevar retos de la misma	• Creación de formatos para la colección de datos, políticas de salud y capacitaciones para profesionales de salud en consejería y en TVT-TA

LÁMINA 2: Teoría 1

Dentro de un sistema de salud jerárquico

➡

Facilitación de discusiones y reflexión en grupo→

Compartir lecciones aprendidas y múltiples perspectivas →

Mayor visualización de brechas/problemas, previo al tamizaje (por ej. la falta de seguimiento) →

Acuerdo sobre los objetivos ideales del programa de tamizaje

➡

Adopción y mantenimiento del programa

LÁMINA 3: Teoría 2

Había un alto índice de pérdida de seguimiento en el nivel secundario de atención.

➡

Traslado de las tareas de tamizaje y tratamiento del VPH al nivel primario→

Reducción de las barreras impuestas por la estrategia anterior

➡

Mayores tasas de tamizaje y seguimiento

LÁMINA 4: Teoría 3

Los sistemas de M & E de datos son intensivos y frecuentemente desconectados de otros sistemas (por ej. dificultad de conexión entre datos de hospitales y EESS)

➡

Cambios que se hicieron al sistema de M & E de datos →

Aumento de la habilidad de los proveedores de salud de visualizar el funcionamiento del sistema de salud →

Mayor oportunidad de seguir a las pacientes al largo de todo el proceso de su cuidado

➡

Aumento en la tasa de tratamiento y una reducción en las tasas de pérdida de seguimiento

LÁMINA 5: Teoría 4

Frecuentes impactos sistémicos como cambios políticos o la pandemia COVID-19

→

Colaboración con campeones/aliados del programa →

Compromiso con el programa a largo plazo→

Continuidad de relaciones y comunicación entre colaboradores y resolución de problemas a nivel local

→

Mayor resiliencia y sostenibilidad de seguimiento
