# Supplementary material for: A realist evaluation of a novel cervical cancer prevention strategy in Iquitos, Peru
Source: PLOS Glob Public Health. 2025 Nov 19;5(11):e0004517. doi: 10.1371/journal.pgph.0004517 (PMC12629488; doi:10.1371/journal.pgph.0004517)
Supplement: S2 Text — (PDF) [file pgph.0004517.s002.pdf]

**“Developing and improving strategies for cervical cancer prevention in Peru”**

## <u>Interview guide for post-implementation evaluation with collaborators</u>

*At the beginning of each interview, participants will give their informed consent. If the interview is in person, the informed consent will be written, and if it is virtual or by telephone, the consent will be given orally and recorded.*

## I. Introduction

a. *Presentation and explanation of the interview’s objectives.*

*Thank you in advance for your time and availability. As you know, the purpose of this interview is to understand a little more about your perspective on Proyecto Precancer in your area, including the results of the project, the challenges that influenced the project, and your thoughts on how changes in the health system occurred and who was affected by these changes.*
*There are no right or wrong questions or answers—we are only interested in hearing about your experiences and opinions..*
*[Note the role and level of authority of the person interviewed in the health system. For example: "Midwive, XX Health Facility"]*

---

## II. Questions

**A. Achievements**

1. From your perspective, what do you think have been the most important achievements of this HPV screening and management program?
    a. Can you give me an example?
    b. How have these results differed from other projects’ results (if applicable)?

**B. Processes**

2. What was different about this process or way of working?
3. How has this process differed from other projects’ processes?
    a. Other organizations?
    b. Other health system initiatives?
4. Do you think others who would have a different opinion about the most important achievements or processes? If so, why?

5. Throughout the project, various activities were carried out and tools were used during each part of the process. **(PAGE 1: Activities and Tools)**
    a. What was useful or not useful about these processes?
    b. Did anything stand out to you about these processes?
    c. What struck you as different?

6. What lessons learned from this experience would you take away and apply to another program?
    a. Why is that aspect important to replicate?
    b. How would you improve this process?

**C. Program Theories**

We think there are 4 processes that were really important in ensuring the achievements of this program and we would like to know your opinion about our theories. If you disagree with our theories, that’s fine: the information you are giving us will help us to formulate new theories.

7. The first theory that we have is: **(PAGE 2: Theory 1)**
    a. First I want to confirm if you agree that we have a hierarchical health care system. Do you agree? Could you give an example?
    b. What do you think about that theory?
    c. Do you think, for example, that understanding the importance of follow-up influenced the decision to adopt this new screening strategy?
    d. Would you modify this theory in any way?

8. The second theory that we have is: **(PAGE 3: Theory 2)**
    a. First, what do you think about the idea that this health system, before this program was implemented, had a high rate of loss to follow-up and difficulties in data collection?
    b. What do you think about this theory?
    c. Would you modify it in any way?

9. The third theory that we have is: **(PAGE 4: Theory 3)**
    a. First, we want to know what you think about the idea that data-intensive M&E systems are often disconnected from other systems.
    b. What do you think about that theory?
    c. Would you modify that theory in any way?

10. The fourth theory that we have is: **(PAGE 5: Theory 4)**
    a. Finally, in terms of context, do you agree that this health system has had frequent systemic impacts, like frequent political changes, or the COVID-19 pandemic?
    b. What do you think about that theory?
    c. Would you modify it in any way?

**D. Summary**

*In summary, let’s end with three slightly more general questions.*

11. If you could change one thing about this program so that it worked better, what would you change, and why?
12. We mentioned before that this program, or other similar health programs, might be able to work in a different manner in other places. What is special about this project that makes it work or not work?
13. To conclude, what else do you think we need to know to really understand how it has worked in your area?

# PAGE 1: Activities and Tools

| | |
|---|---|
| • Workshops at the beginning of the project; first only midwives, then only physicians, then everyone together | • Visualization of the details of the health care system and continuum of care for women in charts and modeling the impact of each decision |
| • Design workshops to plan implementation and overcome implementation challenges | • Creation of data collection sheets, health policies, and training for health professionals in counseling and visual assessment for treatment and thermal ablation |

# PAGE 2: Theory 1

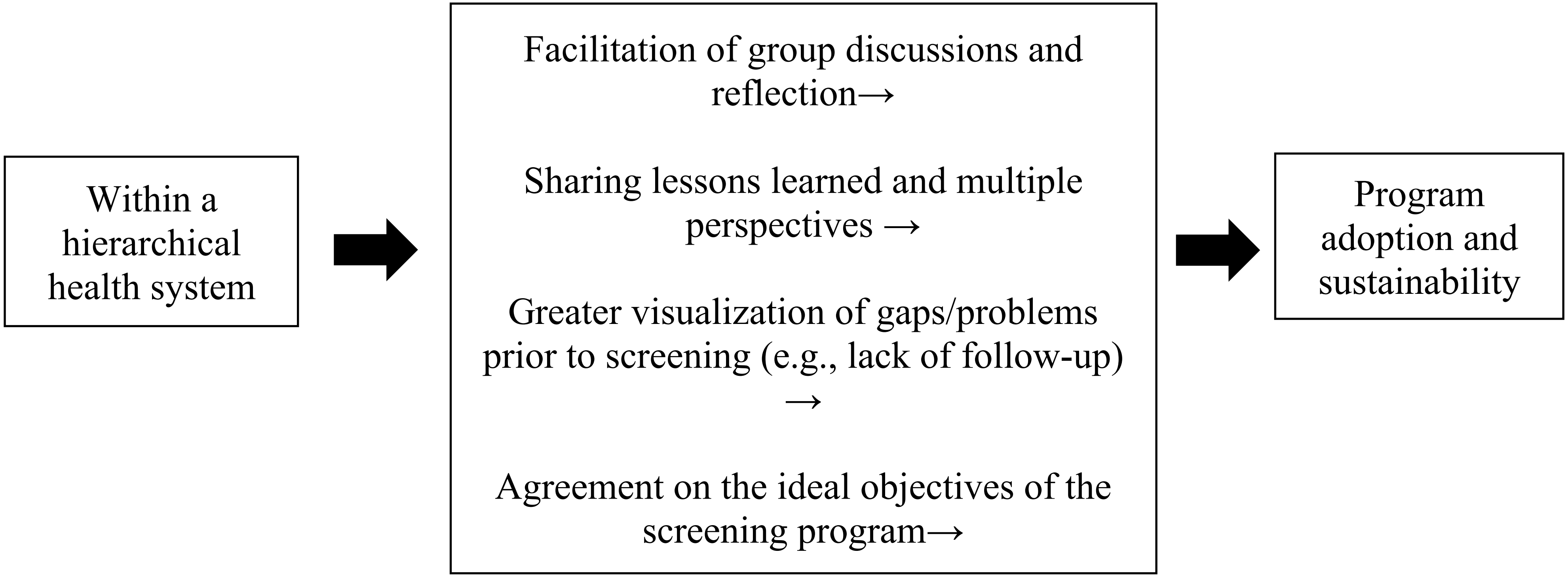

# PAGE 3: Theory 2

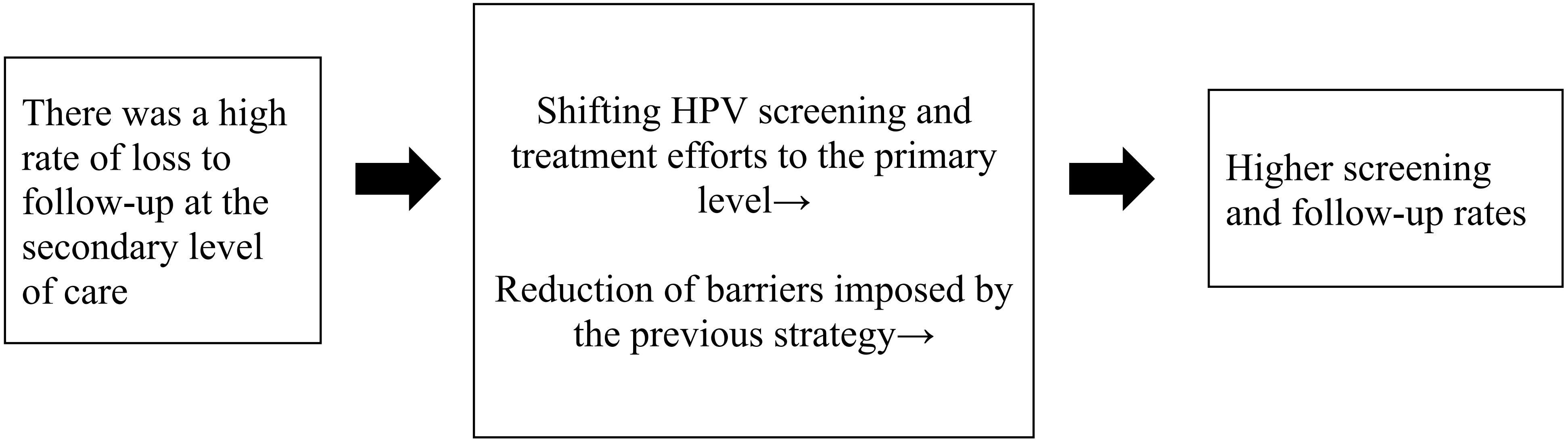

# PAGE 4: Theory 3

Data-intensive M&E systems are often disconnected from other systems (e.g., the difficulty of connecting data between hospitals and health facilities)

➡

Changes made to the M&E data system →

Increased ability of health providers to visualize the functioning of the health system →

Increased opportunity to track patients along the entire continuum of care→

➡

Increased treatment rates and decreased loss-to-follow-up rates

# PAGE 5: Theory 4

Frequent systemic impacts such as political changes or the COVID-19 pandemic

→

Collaboration with program champions/partners →

Commitment to the program over the long term→

Continuity of relationships and communication between collaborators and problema-solving at the local level→

→

Increased program resilience and sustainability
