# Supplementary material for: A realist evaluation of a novel cervical cancer prevention strategy in Iquitos, Peru
Source: PLOS Glob Public Health. 2025 Nov 19;5(11):e0004517. doi: 10.1371/journal.pgph.0004517 (PMC12629488; doi:10.1371/journal.pgph.0004517)
Supplement: S3 Text — (PDF) [file pgph.0004517.s003.pdf]

Resumen

Proyecto Precancer es un proyecto de ciencia de la implementación y pensamiento sistémico cuyo objetivo es facilitar la adopción de una nueva intervención de detección y tratamiento para la prevención del cáncer de cuello uterino en Iquitos, en la cuenca amazónica peruana. Esta intervención introdujo pruebas moleculares del virus del papiloma humano y trasladó el tratamiento del nivel terciario al nivel primario mediante la evaluación visual para el tratamiento y la termoablación para las personas elegibles. Para informar sobre cómo ampliar este proyecto piloto a nuevas regiones de Perú en colaboración con el Ministerio de Salud, llevamos a cabo esta evaluación realista para conocer qué funcionó en nuestra intervención piloto en Iquitos, en qué circunstancias, para quién y por qué. Desarrollamos cuatro teorías iniciales del programa, las probamos mediante entrevistas con 32 partes interesadas, las perfeccionamos basándonos en las entrevistas, y añadimos una quinta teoría. Esta evaluación reveló que la colaboración continua con los proveedores de manera horizontal en todas las jerarquías profesionales de los sistemas (teorías del programa 1, 4 y 5) y la atención centrada en los pacientes con una atención continua más cómoda y accesible pueden, en última instancia, conducir a una mejora de las tasas de detección y tratamiento y a una reducción de la pérdida de pacientes durante el seguimiento (teorías 2 y 3). Además, aprendimos de nuestros colaboradores que la investigación integrada en el sistema de salud pública tenía un alto potencial de sostenibilidad debido a la apropiación local. Estas ideas servirán de base para nuestro trabajo, ya que este proyecto ayuda al Ministerio de Salud a adaptar y ampliar la intervención en otras partes del Perú.
